# Circadian rhythms, Wnt/beta-catenin pathway and PPAR alpha/gamma profiles in diseases with primary or secondary cardiac dysfunction

**DOI:** 10.3389/fphys.2014.00429

**Published:** 2014-11-04

**Authors:** Yves Lecarpentier, Victor Claes, Guillaume Duthoit, Jean-Louis Hébert

**Affiliations:** ^1^Centre de Recherche Clinique, Centre Hospitalier Régional de MeauxMeaux, France; ^2^Department of Pharmaceutical Sciences, University of AntwerpWilrijk, Belgium; ^3^Institut de Cardiologie, Hôpital de la Pitié-SalpêtièreParis, France

**Keywords:** circadian rhythms, Wnt/beta-catenin, PPAR, diabetes, hypertension, myocardial ischemia, neurodegenerative diseases, colon cancer

## Abstract

Circadian clock mechanisms are far-from-equilibrium dissipative structures. Peroxisome proliferator-activated receptors (PPAR alpha, beta/delta, and gamma) play a key role in metabolic regulatory processes, particularly in heart muscle. Links between circadian rhythms (CRs) and PPARs have been established. Mammalian CRs involve at least two critical transcription factors, CLOCK and BMAL1 (Gekakis et al., 1998; Hogenesch et al., 1998). PPAR gamma plays a major role in both glucose and lipid metabolisms and presents circadian properties which coordinate the interplay between metabolism and CRs. PPAR gamma is a major component of the vascular clock. Vascular PPAR gamma is a peripheral regulator of cardiovascular rhythms controlling circadian variations in blood pressure and heart rate through BMAL1. We focused our review on diseases with abnormalities of CRs and with primary or secondary cardiac dysfunction. Moreover, these diseases presented changes in the Wnt/beta-catenin pathway and PPARs, according to two opposed profiles. Profile 1 was defined as follows: inactivation of the Wnt/beta-catenin pathway with increased expression of PPAR gamma. Profile 2 was defined as follows: activation of the Wnt/beta-catenin pathway with decreased expression of PPAR gamma. A typical profile 1 disease is arrhythmogenic right ventricular cardiomyopathy, a genetic cardiac disease which presents mutations of the desmosomal proteins and is mainly characterized by fatty acid accumulation in adult cardiomyocytes mainly in the right ventricle. The link between PPAR gamma dysfunction and desmosomal genetic mutations occurs via inactivation of the Wnt/beta-catenin pathway presenting oscillatory properties. A typical profile 2 disease is type 2 diabetes, with activation of the Wnt/beta-catenin pathway and decreased expression of PPAR gamma. CRs abnormalities are present in numerous pathologies such as cardiovascular diseases, sympathetic/parasympathetic dysfunction, hypertension, diabetes, neurodegenerative diseases, cancer which are often closely inter-related.

## Introduction

CRs are biological temporal processes that display endogenous, entrainable free-running periods that last approximately 24 h. They are driven by molecular internal clocks which can be reset by environmental light-dark cycles. Circadian clocks are transcriptionally based molecular mechanisms which comprise feedback loops (Edery, 2000). The molecular basis of CRs have first been clarified in Drosophila and Neurospora, then in cyanobacteria, plants, and mammals (Reppert and Weaver, 2002). All living organisms adjust their physiology and behavior to the 24-h day-night cycle under the governance of circadian clocks. Circadian clocks may provide a selective advantage of anticipation, thus allowing organisms to respond efficiently to various stimuli at the appropriate time. In mammals, sleep-awake and feeding patterns, hormone secretion, heart rate, blood pressure, energy metabolism, and body temperature exhibit CRs. Their disruptions may have deleterious effects. People submitted to shift working, frequent transmeridian air flight, exposure to artificial light exhibit a particularly high incidence of metabolic syndrome and obesity. CR dysfunctions in blood pressure and heart rate, which are both partly regulated by PPAR gamma are involved in arrhythmias which may lead to sudden cardiac death, myocardial infarction or stroke, often occurring at the early morning during the surge in blood pressure. CRs are dissipative structures due to a negative feedback produced by a protein on the expression of its own gene (Goodwin, 1965; Hardin et al., 1990). They operate far-from- equilibrium and generate order spontaneously by exchanging energy with their external environment (Prigogine et al., 1974; Goldbeter, 2002; Lecarpentier et al., 2010).

## The regulatory sites of circadian rhythms

The master regulator site of CRs is the suprachiasmatic nucleus (SCN) inside the hypothalamus in which core clock genes are rhythmically expressed (Weaver, 1998). In addition to this central clock, each organ has its own biological clock system, termed peripheral clock. The SCN and most peripheral tissues such as heart, blood vessels, skeletal muscles, kidneys, liver, and fat, govern numerous functions that are synchronized with the sleep-awake cycle (Zylka et al., 1998). In the cardiovascular system, circadian clocks have been described within numerous mammalian cells, such as cardiomyocytes, vascular smooth muscle cells, endothelial cells, and fibroblasts (McNamara et al., 2001; Nonaka et al., 2001; Durgan et al., 2005, 2007; Takeda et al., 2007). Peripheral clocks have their own regulatory mechanisms, which are specific to each peripheral organ by regulating the expression of clock-controlled genes (*Ccg*). CRs have been demonstrated in approximately 8–10% of total genes expressed in mouse heart and liver, more than 90% of them depending on self-autonomous local diurnal oscillators (Storch et al., 2002).

## Genes and proteins

Important genes are involved in CRs including *Clock* (*Circadian locomotor output cycles kaput*), *Bmal1* (*brain and muscle aryl-hydrocarbon receptor nuclear translocator-like 1*), *Cry1* (*cryptochrome 1*), *Cry2* (*cryptochrome 2*), *Per1* (*Period 1*), *Per2* (*Period 2*), *Per3* (*Period 3*), and *Ccg*. They organize transcription/translation autoregulatory feedback loops comprising both activating and inhibiting pathways (Reppert and Weaver, 2002; Schibler and Sassone-Corsi, 2002). A complex network is formed by all these genes which interlock feedback and forward subtle loops whose complete time course is approximately 24 h. Clock genes Per1, Per2, Bmal1, and Cry1 display rhythmic expression in human hearts (Leibetseder et al., 2009). At the start of the day, transcription of Clock and Bmal1 begins. The proteins CLOCK and BMAL1 are synthesized and then associate as dimers which bind to regulatory DNA sequences (E-box elements) of the promoters of target genes. CLOCK: BMAL1 dimer activates circadian gene transcription of Period genes (Per1, 2, and 3), Chryptochrome genes (Cry 1 and 2), Rev-erb, Ror (related orphan receptor), and Ccg, and drives their rhythmic expression (Reppert and Weaver, 2001, 2002; Young and Kay, 2001; Canaple et al., 2006; Chen and Yang, 2014). Into the cytoplasm, the PER and CRY proteins dimerize and, after translocation to the nucleus, modulate the transcriptional activity of CLOCK: BMAL1 (Kume et al., 1999). Concentrations of BMAL1 and PER proteins cycle in counterpoint. PER2 is a positive regulator of the Bmal1 loop. Protein CRY is a negative regulator of both Per and Cry loops. ROR alpha enhances Bmal1 transcription (Akashi and Takumi, 2005), while the nuclear receptor Rev-erb inhibits it (Ueda et al., 2002). Rev-erb alpha binds to ROR-responsive element (RORE) in the Bmal1 promoter and represses its transcriptional activity (Preitner et al., 2002). Rev-erb alpha protein is a member of the nuclear receptor family of intracellular transcription factors. The gene Rev-erb alpha is a major regulatory component of the circadian clock (Yin et al., 2006) and among various properties, is involved in the circadian expression of plasminogen activator inhibitor type 1 (Wang et al., 2006).

### Circadian rhythms and mutations of genes

Mutations or deletions of clock genes in mice have shown the key role of circadian clocks to ensure the proper timing of metabolic and cardiovascular processes. There is an increased pathological remodeling and vascular injury together with an aberrant CR in *Bmal1*-knockout and *Clock* mutant mice (Anea et al., 2009). Aortas from *Bmal1*-knockout and *Clock* mutant mice exhibit endothelial dysfunction. Akt (protein kinase B) and subsequent nitric oxide signaling is significantly attenuated in arteries from *Bmal1*-knockout mice. *Bmal1* is a key regulator of myogenesis which may represent a temporal regulatory mechanism to fine-tune myocyte differentiation (Chatterjee et al., 2013). *Bmal1* regulates adipogenesis *via the* Wnt signaling pathway (Guo et al., 2012). Disruption of *Bmal1* in mice led to increased adipogenesis, adipocyte hypertrophy, and obesity. Attenuation of *Bmal1* function resulted in down-regulation of genes in the canonical Wnt pathway known to suppress adipogenesis. Promoters of these genes, i.e., *beta-catenin*, Disheveled *(Dsh)*, T cell-enhancing binding *(Tcf)* display *Bmal1* occupancy, indicating direct circadian regulation by Bmal1. Among several abnormalities, deletion of the clock gene *Bmal1* in mice adipose tissue induces obesity (Paschos et al., 2012). The cardiomyocyte-specific clock mutant *(Ccm)* is a mouse model wherein the cardiomyocyte circadian clock is selectively suppressed (Young et al., 2001b,c; Durgan et al., 2006). *Ccm* presents a temporal suspension of the cardiomyocyte circadian clock at the wake-to-sleep transition (Young, 2009). Numerous mutations of genes will be discussed in the following paragraphs of this review.

### Circadian rhythms and heart performance

Loss of synchronization between the internal clock and external stimuli can induce cardiovascular organ damage. Discrepancy in the phases between the central and peripheral clocks also seems to contribute to progression of cardiovascular disorders (Takeda and Maemura, 2011). Peripheral clocks have their own roles specific to each peripheral organ by regulating the expression of *Ccg*, although the oscillation mechanisms of the peripheral clock are similar to that of the SCN (Takeda et al., 2007). Both the physiological and pathological functions of cardiovascular organs are closely related to CRs. Heart rate, blood pressure and endothelial function show diurnal variations within a day. A profound pattern exists in the time of day at which the death may occur (Takeda and Maemura, 2011). The onset of cardiovascular disorders such as acute coronary syndrome, atrial arrhythmias, and subarachnoid hemorrhage exhibits impairment of diurnal oscillations. Stroke and heart attacks most frequently happen in the morning when blood pressure surges.

Over the course of the day, the normal heart anticipates, responds and adapts to physiological alterations within its environment. Contractile performance, carbohydrate oxidation, fatty acid oxidation (FAO), mitochondrial function, oxygen consumption, and expression of all metabolic genes show diurnal variations. The circadian clock plays an important role in cardiac homeostasis through the anticipation of daily workload. In wild-type mice, the ejection fraction (EF) and the shortening fraction (FS) show circadian variation (Wu et al., 2011). The diurnal variations in EF and FS are altered in mice with disruptions of circadian clock genes and are significantly diminished under an imposed light regimen. The circadian variation in blood pressure and heart rate is disrupted in *Bmal1*(-/-) and *Clock* (mut) mice in which core clock genes are deleted or mutated (Curtis et al., 2007). *Bmal1* deletion abolishes the 24-h frequency in cardiovascular rhythms. However, a shorter ultradian rhythm remains. Sympathetic adrenal function is disrupted in these mice.

### Peroxisome proliferator-activated receptors (PPARs)

PPARs (alpha, beta/delta, and gamma) are nuclear receptors belonging to the nuclear receptor superfamily. They function as transcription factors within the cell nuclei and regulate the expression of several target genes. PPARs play a pivotal role in various physiological and pathological processes, especially in energy metabolism, development, carcinogenesis, extracellular matrix remodeling, and CRs (Lockyer et al., 2009). PPARs heterodimerize with the retinoid X receptor (RXR). PPARs are activated by their respective ligands, either endogenous fatty acids or pharmaceutical drugs which are potential therapeutic agents. Numerous natural and synthetic compounds, i.e., fatty acids, eicosanoids, arachidonic acid, hypolipidemic fibrates activating PPAR alpha, and anti diabetic thiazolidinediones (TZD) activating PPAR gamma, serve as activators of PPARs. PPARs are involved in numerous pathologies such as obesity, dyslipidemia, insulin resistance, type 2 diabetes, hypertension, cardiac hypertrophy (Berger and Moller, 2002; Kelly, 2003). PPAR beta/delta was not studied in this review.

### PPARs and circadian rhythms

PPARs integrate the mammalian clock and energy metabolism (Chen and Yang, 2014). PPARs have been shown to be rhythmically expressed in mammalian tissues (Yang et al., 2006) and to directly interact with the core clock genes (Inoue et al., 2005). PPAR beta/delta has not been studied in this review.

#### PPAR alpha

PPAR alpha presents CRs in several organs, i.e. heart, muscles, liver, and kidney (Lemberger et al., 1996; Yang et al., 2006). PPAR alpha expression is stimulated by stress, glucocorticoid hormones, and insulin whose secretion follows CRs (Lemberger et al., 1994). Importantly, PPAR alpha is a direct target of genes (*Bmal1* and *Clock*) through an E-box process (Oishi et al., 2005). The circadian expression of PPAR alpha mRNA is abolished in the liver of homozygous *Clock* mutant mice and is regulated by the peripheral oscillators in a CLOCK-dependent mechanism. In rodent liver, there is a regulatory feedback loop involving BMAL1 and PPAR alpha in peripheral clocks. This regulation occurs via a direct binding of PPAR alpha on a PPAR alpha response element located in the *Bmal1* gene promoter. Moreover, BMAL1 is an upstream regulator of *PPAR alpha* gene expression (Gervois et al., 1999; Canaple et al., 2006). Several genes such as those encoding for sterol regulatory element binding protein, HMG-CoA reductase, fatty acid synthase, are involved in the lipid metabolism. They display circadian fluctuations, and their activities are diminished or suppressed in *PPAR alpha* knockout mice (Patel et al., 2001; Gibbons et al., 2002). PPAR alpha directly regulates the transcriptional activity of *Bmal1* and *Rev-erb alpha* through the PPRE located in the promoter site of their respective genes. *Per2* interacts with nuclear receptors including PPAR alpha and Rev-Erb alpha and serves as a co-regulator of nuclear receptor-mediated transcription. The PPAR alpha agonist fenofibrate increases transcription and resets circadian expression of *Bmal1, Per2, and Rev-erb alpha* in mouse liver (Canaple et al., 2006) and cultured hepatocytes (Gervois et al., 1999). Moreover, bezafibrate can phase advance the rhythmic expression of *Bmal1, Per2, and Rev-erb alpha* in several mouse peripheral tissues (Shirai et al., 2007; Oishi et al., 2008).

#### PPAR gamma

PPAR gamma exhibits variations in diurnal expression in mouse fat, liver, and blood vessels (Yang et al., 2006; Wang et al., 2008). Deletion of *PPAR gamma* in mouse suppresses or diminishes diurnal rhythms (Yang et al., 2012). CRs have been analyzed in two strains of whole-body *PPAR gamma null* mouse models, i.e., *Mox2-Cre* mice (*MoxCre/flox*) or induced by tamoxifen (*EsrCre/flox/TM*). Diurnal variations in blood pressure and heart rate are blunted *in MoxCre/flox* mice. Impaired rhythmicity of the canonical clock genes is observed in adipose tissue and liver. This shows the important role of PPAR gamma in the coordinated control of circadian clocks, metabolism, and cardiac performance (Yang et al., 2012). Moreover, insulin resistance is correlated with a non-dipper type—i.e., with no blood pressure decrease during the circadian cycle- in essential hypertension. TZD are oral hypoglycemic agents act as insulin sensitizers and possess antihypertensive properties. TZD therapy with pioglitazone transforms the CR of blood pressure from a non-dipper to a dipper type (Anan et al., 2007). PPAR gamma contributes to maintain the diurnal variations of both blood pressure and heart rate.

Rev-Erb alpha, an orphan nuclear receptor and a core clock component, is expressed after PPAR gamma activation with rosiglitazone in rat. Activated PPAR gamma induces *Rev-Erb alpha* promoter activity by binding to the response element *Rev-DR2*. Mutations of the 5′ or 3′ half-sites of the response element suppress PPAR gamma binding and transcriptional activation (Fontaine et al., 2003). PGC-1 alpha, a transcriptional co-activator that regulates energy metabolism, is rhythmically expressed in the liver and skeletal muscle of mice. PGC-1 alpha stimulates the expression of clock genes, notably *Bmal1* and *Rev-erb alpha*, through co-activation of the ROR family of orphan nuclear receptors. Mice lacking PGC-1 alpha show abnormal CRs of activity, body temperature, and metabolic rate (Liu et al., 2007a). *Nocturnin*, a circadian-regulated gene, promotes adipogenesis by stimulating PPAR gamma nuclear translocation (Kawai et al., 2010). Nocturnin binds to PPAR gamma and stimulates its transcriptional activity whereas its deletion suppresses PPAR gamma oscillations (Green et al., 2007). The hormone-dependent interaction of the nuclear receptor RXR alpha with CLOCK negatively regulates CLOCK: BMAL1-mediated transcriptional activation of clock gene expression in vascular cells. RXR alpha can phase shift Per2 mRNA rhythmicity, providing a molecular mechanism for hormonal control of clock gene expression (McNamara et al., 2001).

### Canonical Wnt/beta-catenin pathway

**Figure 1:**
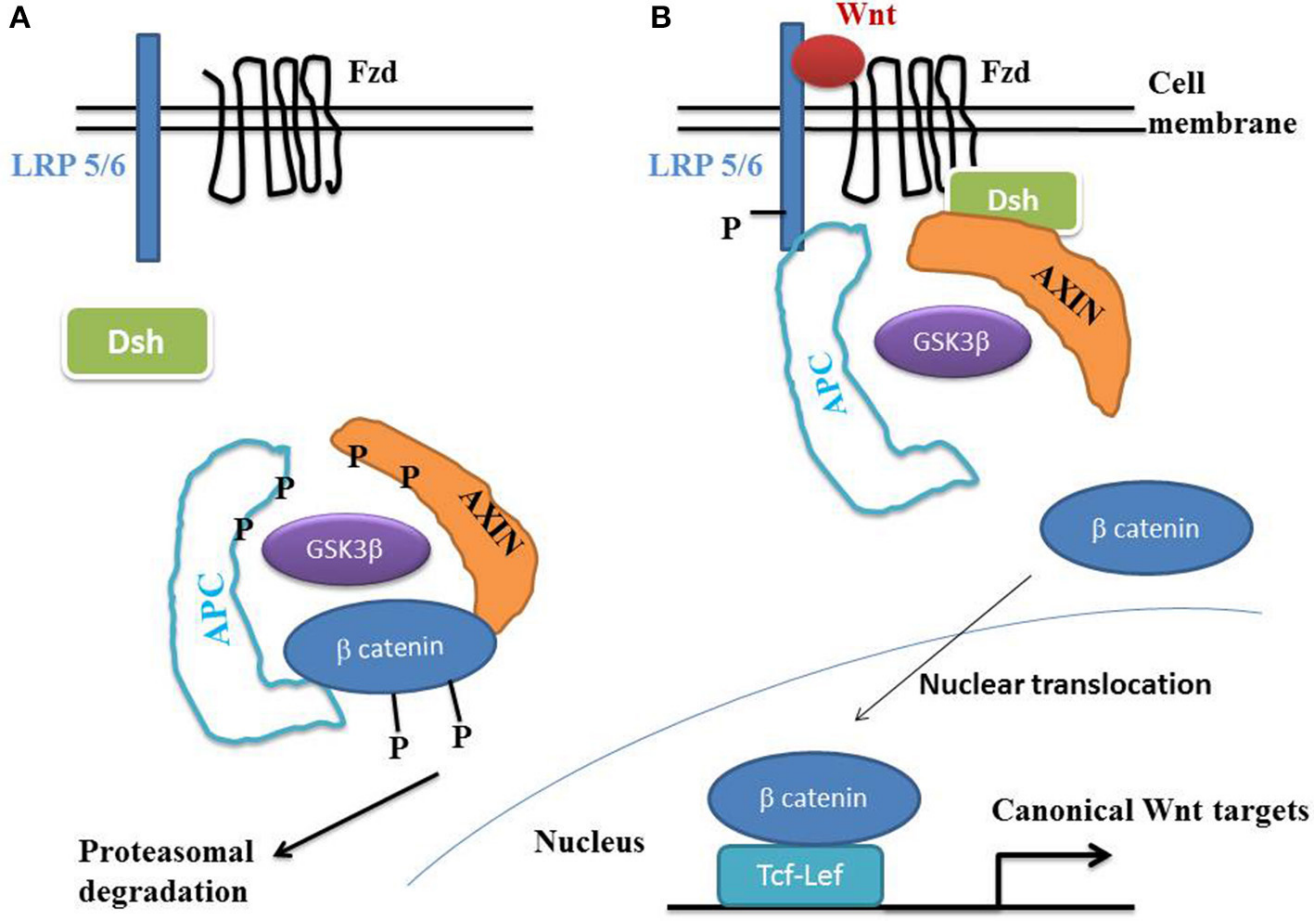
**The Wnt/beta-catenin pathway. (A)** In the absence of Wnt, cytosolic beta-catenin is phosphorylated by GSK3 beta. APS and AXIN complex with GSK3 beta and beta-catenin to enhance the destruction process into the proteasome. Phosphorylated beta-catenin is recognized by the ubiquitin ligase beta -TrCP, ubiquinated and degraded. The Wnt pathway is in an “off state.” **(B)** In the presence of Wnt, Wnt binds both Frizzled and LRP5/6 receptors to initiate GRK5/6-mediated LRP phosphorylation and disheveled-mediated Frizzled internalization. Disheveled membrane translocation leads to dissociation of the AXIN/APC/GSK3 beta complex. Beta-catenin phosphorylation is inhibited and accumulates into the cytosol. Beta-catenin then translocates to the nucleus to bind Lef-Tcf co-transcription factors, which induces the Wnt-response gene transcription. Abbreviations: APC, adenomatous polyposis coli; Dsh, Disheveled; GSK3 beta, glycogen synthase kinase 3 beta; LRP5/6, low density lipoprotein receptor-related protein 5/6; Fzd, Frizzled.

Beta-catenin plays a key role during epithelial-mesenchymal transition (EMT), that characterizes normal embryonic development, tissue regeneration and cancer proliferation (Heuberger and Birchmeier, 2010). Beta-catenin is a normal constituent of the zonula adherens, a major cell-to-cell adhesion complex in pavement-like tissues. During EMT, the loss of cadherins disrupts the zonula adherens, thus liberating beta-catenin into the cytoplasm. This molecule then migrates to the cell nucleus where it activates the Wnt/beta-catenin target genes. A hallmark of the canonical Wnt pathway activation is the elevation of cytoplasmic beta-catenin protein levels, the subsequent nuclear translocation and further activation of beta-catenin specific gene transcription (Ben-Ze'ev and Geiger, 1998; Klymkowsky et al., 1999; Zhurinsky et al., 2000; Moon et al., 2002; Maeda et al., 2004; Sen-Chowdhry et al., 2005; Garcia-Gras et al., 2006), (Figure 1). In the absence of Wnt ligands, beta-catenin is recruited into a destruction complex that contains adenomatous polyposis coli (APC) and Axin, which facilitate the phosphorylation of beta-catenin by glycogen synthase kinase 3- beta (GSK3-beta). GSK3-beta phosphorylates the N-terminal domain of beta-catenin, thereby targeting it for ubiquitination and proteasomal degradation. In the presence of a Wnt ligand, the binding of Wnt to Frizzled (Fzd) leads to activation of the phosphoprotein Disheveled (Dsh). Dsh recruits Axin and the destruction complex to the plasma membrane, where Axin directly binds to the cytoplasmic tail of LRP5/6. Axin is degraded, which decreases beta-catenin degradation. The activation of Dsh also leads to the inhibition of GSK3-beta by phosphorylation, which further reduces the phosphorylation and degradation of beta-catenin. The beta-catenin degradation complex is inactivated with recruitment of axin to the plasma membrane, thus stabilizing the non-phosphorylated beta-catenin which translocates to the nucleus. Beta-catenin binds to T cell/lymphoid-enhancing binding (Tcf/Lef) transcription factors. The resulting complex becomes active by displacing Grouchos, leading to activation of numerous target genes.

### Canonical Wnt/beta-catenin pathway and PPAR gamma

Numerous studies have shown the direct interaction between beta-catenin and PPAR gamma (Moldes et al., 2003; Jansson et al., 2005; Garcia-Gras et al., 2006). PPAR gamma activation inhibits the beta-catenin activation of Tcf/Lef transcription factors (Lu and Carson, 2010). The TZD PPAR gamma agonists troglitazone, rosiglitazone, and pioglitazone, and the non-TZD PPAR gamma activator GW1929 inhibit the beta-catenin-induced transcription in a PPAR gamma dependent manner. Activation of the Wnt-beta catenin pathway leads to osteogenesis, not adipogenesis and its inhibition leads to an increase in transcription of PPAR gamma. Osteogenic pathway is linked to the stimulation of Wnt signal leading to the final transcriptional activation of early osteogenic markers such as RUNX-2 and ALP, mediated by beta-catenin. Conversely, the adipogenic pathway involves inhibition of Wnt pathway leading to ubiquitination/degradation of beta-catenin which results in the transcription of PPAR gamma, a pivotal initiator of adipogenesis. The canonical Wnt/beta-catenin-PPAR gamma system determines the molecular switching of osteablastogenesis vs. adipogenesis (Takada et al., 2009). PPAR gamma is a prime inducer of adipogenesis that inhibits osteoblastogenesis. Two different pathways switch the cell fate decision from adipocytes to osteoblasts by suppressing the transactivation function of PPAR gamma. TNF-alpha- and IL-1-induced TAK1/TAB1/NIK signaling cascade attenuate PPAR gamma-mediated adipogenesis by inhibiting the binding of PPAR gamma to the DNA response element. PPAR gamma suppresses Wnt/beta-catenin signaling during adipogenesis (Moldes et al., 2003). Wnt/beta-catenin pathway operates to maintain the undifferentiated state of preadipocytes by inhibiting adipogenic gene expression. Importantly, there is a reciprocal relationship between beta-catenin expression and PPAR gamma activity.

### Diseases associated with deactivation of the Wnt/beta-catenin pathway and increased expression of PPAR gamma

Numerous diseases present a common denominator: activation of the Wnt/beta-catenin pathway decreased and the expression of PPAR gamma increased. In most cases, expression of PPAR alpha decreases.

#### Arrhythmogenic right ventricular cardiomyopathy (ARVC)

**Figure 2:**
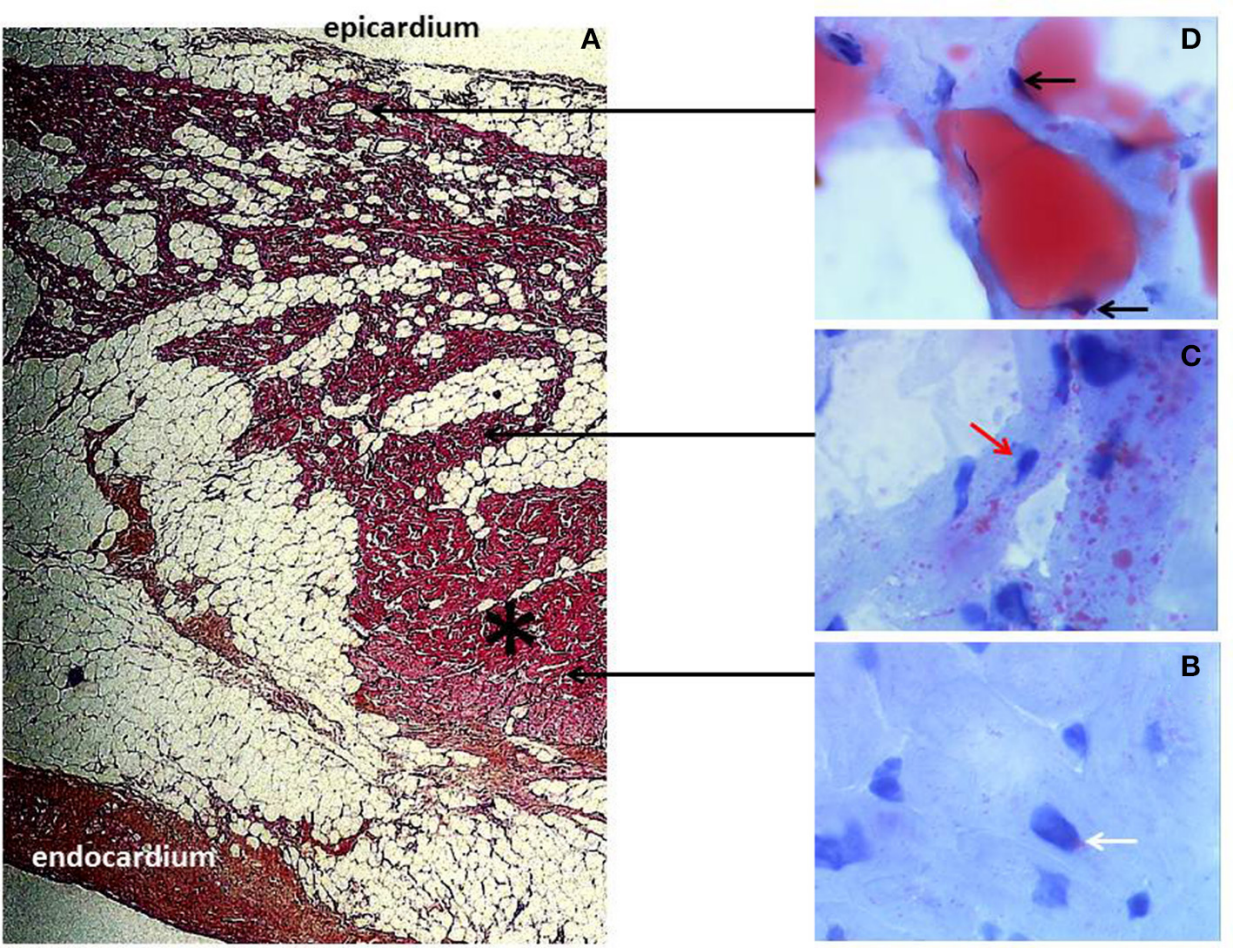
**Arrhythmogenic right ventricular cardiomyopathy (ARVC) histology. (A)** Typical morphology of right ventricular transmural free wall section in a terminal ARVC heart transplant specimen, showing extensive fibro-fatty replacement. A mid-mural residual muscular core (black asterisk) is well-identified. Fibrosis is prominently located at the subendocardium. Note the layer of normal subepicardial fat (Hematoxylin Eosin Saffron staining, original magnification × 10). **(B–D)** are fresh tissue snap frozen fragments representative of regions referred to as muscular **(B,C)** and fatty myocardium, **(D)** respectively, stained with oil red O (original magnification × 50). The red staining indicates neutral lipid accumulation. **(B)** Note the normal discrete perinuclear staining of the cardiomyocytes (white arrow) within the well-preserved myocardial core. **(C)** In contrast, there is an abnormal major accumulation of fatty droplets (not visible under standard staining) dispersed within the cells of the mid mural muscular zone located above the residual muscular core and surrounded by fatty tissue. Note the remaining normal central position of the nucleus within the myocardial cells (red arrow). **(D)** Finally, there is a direct transdifferentiation of myocardial cells into adipocytes within the upper muscular zone bordering the normal subepicardial fat. Take notice of the major confluence of the fatty droplets as well as of the final aspect of total fatty transformation with migration of the nucleus beneath the cell membrane (black arrows).

ARVC is a rare human disease characterized by the development of a fibro-fatty tissue in both ventricles, prominently involving the right ventricular (RV) myocardium (Marcus et al., 1982; Fontaine et al., 1999) (Figure 2). Cardiac dysfunction progressively develops, initially located at the RV and becoming biventricular in about 20% of cases (Richardson et al., 1996; Hebert et al., 2004). ARVC is most often an autosomic family-related disease. Genetic mutations have been identified in about 50% of cases, occurring among the five desmosomal proteins so far identified in the ventricular cardiomyocyte, i.e., desmoglein 2 (DSG2), desmocollin 2 (DSC2), plakophilin 2 (PKP2), plakoglobin (PG), and desmoplakin (DSP) (Basso et al., 2009; Fressard et al., 2010). PPAR abnormalities have been reported in ARVC with an increase in PPAR gamma and a decrease in PPAR alpha in RV (Djouadi et al., 2009).

Molecular mechanisms underlying ARVC are now better understood. The link between PPAR gamma dysfunction and desmosomal genetic mutations implicates the Wnt/beta-catenin pathway. Thus, the suppression of canonical Wnt/beta-catenin signaling by nuclear PG recapitulates the phenotype of ARVD by exhibiting fat accumulation in cardiomyocytes, enhanced myocyte apoptosis, ventricular dysfunction, and ventricular arrhythmias in transgenic mice (Garcia-Gras et al., 2006). The desmosomal PG also known as gamma-catenin, has structural and functional similarities to beta-catenin, which is the effector for canonical Wnt signaling (Moon et al., 2002). PG interacts and competes with beta-catenin at multiple cellular levels with a net negative effect on the canonical Wnt/beta-catenin signaling pathway through Tcf/Lef transcription factors (Ben-Ze'ev and Geiger, 1998; Klymkowsky et al., 1999; Zhurinsky et al., 2000; Maeda et al., 2004). Mutating the desmosomal protein DSP by impairing desmosome assembly set free gamma-catenin from the desmosomes. As a consequence, gamma catenin translocates to the nucleus and after competition with beta-catenin suppresses signaling through the canonical Wnt/beta-catenin-Tcf/Lef pathway. Suppression of DSP expression responsible for human ARVC, leads to nuclear localization of PG and to suppression of canonical Wnt/beta-catenin-Tcf/Lef1 signaling in cultured atrial myocytes and in mouse hearts (Sen-Chowdhry et al., 2005). Tcf/Lef1 suppression induces a transcriptional switch from myogenesis to adipogenesis (Ross et al., 2000). This leads to enhanced adipogenesis, fibrogenesis, and myocyte apoptosis, thus summarizing the phenotype of human ARVC (Corrado et al., 1997).

#### Cardiac hypoxia

Hypoxia up-regulates expression of *PPAR gamma angiopoietin-related* gene in cardiomyocytes (Belanger et al., 2002). Hypoxia-inducible factor 1 alpha (HIF1 alpha) inhibits PPAR alpha expression during hypoxia (Narravula and Colgan, 2001). Hypoxia leads to activation of HIF1 alpha (Krishnan et al., 2009) and several genes involved in the regulation of glucose transporters, glycolytic enzyme, and pyruvate deshydrogenase kinase (PDK1). HIF1 alpha overexpression *in vitro* leads to triacylglycerol accumulation, and reduced FAO due to inhibition of PPAR alpha. Cardiac hypoxia represents a pathological state where expression of PPAR alpha is reduced whereas that of PPAR gamma is increased. Hypoxia triggers a cascade of cellular metabolic responses including a decrease in mitochondrial oxidative flux (Huss et al., 2001). Under hypoxic conditions, myocytes exhibit significant accumulation of intracellular neutral lipid consistent with reduced carnitine palmitoyltransferase-1 (CPT-1) activity and diminished FAO capacity. Hypoxia reduces PPAR alpha/RXR binding activity and had no effect on the nuclear level of PPAR alpha protein. Hypoxia reduced the nuclear and cellular RXR levels and deactivates PPAR alpha by reducing the availability of its obligate partner RXR. In rat models of systemic hypoxia (Razeghi et al., 2001), cardiac hypoxia induces a decrease in heart muscle transcript levels of *PPAR alpha* and *PPAR alpha-regulated* genes (PDK4), muscle CPT-1, and malonyl-CoA decarboxylase. This explains the increased reliance of the heart for glucose during hypoxia.

PPAR gamma co-activator 1 alpha (PGC-1 alpha) is a major regulator of mitochondrial biogenesis and activity in the cardiac muscle. Hypoxia stimulates the expression of PGC-1 alpha in cardiac myocytes (Zhu et al., 2010). PGC-1 alpha stimulates the expression of clock genes, particularly *Bmal1* and *Rev*-*erb alpha* (Liu et al., 2007a). Mice lacking PGC-1alpha present abnormal diurnal rhythms of activity, body temperature, and metabolic rate. Overexpression of PGC-1 alpha inhibits clock gene expression in both heart and skeletal muscles and decreases the expression of PPAR alpha. PGC-1 alpha overexpression abolishes the diurnal variation of EF (Wu et al., 2011) and plays an important role on cardiac function by regulating CRs of metabolic genes.

Ephrins belong to the family of receptor tyrosine kinases. Interestingly, Ephrin-Eph cell signaling is linked to the Wnt/beta catenin pathway (Clevers and Batlle, 2006) and favorably influences cardiomyocyte viability which ultimately preserves cardiac function after myocardial infarction. Ephrin-Eph signaling could potentially be a new therapeutic target in the treatment of myocardial infarction (O'Neal et al., 2013). In non re-perfused hearts of mice with a functional deletion of the CR gene *mPer2*, myocardial infarct size is reduced. A decrease in infarct size in *mPer2-M* mouse hearts following ischemia-reperfusion injury and ischemic preconditioning is observed and improves preservation of myocardial viability. In the *mPer2*-mutant mouse myocardium cardio-protection occurs via the mechanisms connecting cardiac events, mitochondrial function, and *mPer2* (Virag et al., 2013).

#### Cardiac hypertrophy and cardiac overload

Development of cardiac hypertrophy and progression to heart failure induce a change in myocardial metabolism, characterized by a switch from fatty acid utilization to glycolysis, and lipid accumulation. PPAR gamma and HIF-1 alpha are key mediators of lipid anabolism and glycolysis, respectively. They are jointly up-regulated in hypertrophic cardiomyopathy and cooperate to mediate key changes in cardiac metabolism (Krishnan et al., 2009). In response to pathological stress, HIF-1 alpha activates glycolytic genes, and PPAR gamma. This results in increased glycolytic flux, glucose-to-lipid conversion via the glycerol-3-phosphate pathway, and contractile dysfunction. Ventricular deletion of HIF1 alpha in mice prevents hypertrophy-induced PPAR gamma activation, the consequent metabolic re-programming, and contractile dysfunction. HIF-1 alpha and PPAR gamma protein expression is up-regulated in human and mouse cardiac hypertrophy. HIF 1 alpha directly activates PPAR gamma transcription. PPAR gamma is a key downstream effector of HIF-1 alpha-driven triacylglycerol accumulation in cardiomyocytes (Krishnan et al., 2009).

In pathological hypertrophied heart, PPAR alpha expression and activity are diminished, leading to a reduction in the capacity for FAO and increased rate of glucose utilization (Barger et al., 2000). Alpha 1-adrenergic agonist-induced hypertrophy of cardiomyocytes in culture results in a switch in energy substrate preference from fatty acids to glucose and in a significant decrease in palmitate oxidation rates together with a reduction in the expression of the gene encoding muscle carnitine palmitoyltransferase 1 (*M-CPT1*). Cardiac myocyte transfection has shown that *M-CPT1* promoter activity is repressed during cardiomyocyte hypertrophic growth, an effect involving a *PPAR alpha* response element. Hypertrophied myocytes exhibited reduced capacity for cellular lipid homeostasis, as evidenced by intracellular fat accumulation. Thus, during cardiomyocyte hypertrophic growth, PPAR alpha is deactivated at several levels, leading to diminished capacity for myocardial lipid metabolism. The functional consequences of this metabolic switch from lipid to glucose may serve to preserve ventricular function in the context of chronic pressure overload (Young et al., 2001a). During cardiac pressure overload-induced cardiac hypertrophy, the diurnal variation of metabolic gene expression is completely suppressed and the cardiac performance is impaired (Young et al., 2001b). The induction of clock output genes is attenuated in the pressure-overloaded hypertrophied heart, providing evidence for a diminished ability of the hypertrophied heart to anticipate and subsequently to adapt to physiological alterations during the day (Young et al., 2001c).

#### Osteoporosis

The Wnt pathway induces differentiation of bone-forming cells (osteoblasts) and suppresses the development of bone-resorbing cells (osteoclasts). It is controlled by antagonists that interact either with Wnt proteins (Wnts) or with Wnt co-receptors. Wnts function as key regulators in osteogenic differentiation of mesenchymal stem cells and bone formation. Aberrant Wnt pathways are associated with many osteogenic diseases (Rawadi and Roman-Roman, 2005; Canalis, 2013). Both human genetics and animal studies have pointed out the role of the Wnt/LRP5 pathway as a major regulator of bone mass. In mice, down-regulation or neutralization of Wnt antagonists enhances bone formation. Mutations in *LRP5* cause primary osteoporosis by reducing Wnt signaling activity and result in decreased bone formation (Korvala et al., 2012). Heterozygous PPAR gamma-deficient mice exhibit high bone mass by stimulating osteoblastogenesis from bone marrow progenitors. Inhibition of PPAR gamma increases osteoblastogenesis and bone mass in male C57BL/6 Mice (Duque et al., 2013). PPAR gamma inhibits osteoblast differentiation (Wan et al., 2007).

Cardiovascular disease and osteoporosis are common age-related conditions associated with significant morbidity and mortality. An increasing body of biological and epidemiological evidences provides support for a link between cardiovascular disease and osteoporosis that cannot be explained by age alone (Farhat and Cauley, 2008). Several hypotheses have been proposed to explain the link between osteoporosis and cardiovascular disease including shared risk factors, common pathophysiological mechanisms and common genetic factors.

#### Alzheimer disease (AD)

AD is a progressive neurodegenerative disorder, neuropathologically characterized by amyloid-beta (Abeta) plaques, and hyperphosphorylated tau accumulation with hereditary missense mutations in the amyloid precursor protein or presenilin-1 and -2 (*PSEN1* and *PSEN2*) genes. Presenilins are involved in modulating beta-catenin stability; therefore familial AD-linked PSEN-mediated effects can reduce the Wnt pathway (Boonen et al., 2009). Tau phosphorylation is mediated by GSK-3 beta, a key antagonist of the Wnt pathway. Sustained loss of function of Wnt/beta-catenin signaling underlies the onset and progression of AD (Inestrosa and Toledo, 2008; De Ferrari et al., 2014). Downregulation of Wnt signaling induced by Abeta is associated with AD progression. Persistent activation of Wnt signaling through Wnt ligands, or inhibition of negative regulators of Wnt signaling, such as Dickkopf-1 and GSK-3 beta are able to protect against Abeta toxicity and ameliorate cognitive performance in AD (Wan et al., 2014). A relationship between amyloid-beta-peptide -induced neurotoxicity and a decrease in the cytoplasmatic levels of beta-catenin has been observed. Although PPAR gamma is elevated in the brain of AD individuals (Jiang et al., 2008), activation of the Wnt signaling pathway may be proposed as a therapeutic target for the treatment of AD.

In old mice engineered to lack *Bmal1*, there is evidence of brain cell damage that looked similar to that seen in AD. BMAL1 in a complex with CLOCK regulates cerebral redox homeostasis and connects impaired clock gene function to neurodegeneration (Musiek et al., 2013). Altered CR synchronization has been reported in the brain of AD patients (Cermakian et al., 2011). CR disturbances affect as many as a quarter of AD patients. Alterations in the SCN and melatonin secretion are the major factors linked with CR abnormalities. Daytime agitation, night-time insomnia, and restlessness are among the common behavioral alterations observed in AD. Normally, in the interstitial fluid, Abeta has a diurnal fluctuation with low levels during sleep and peak levels during wake. Prolonged wake and/or orexin administration increase levels of the Abeta in the interstitial fluid of the brain in mice. Orexin antagonist reduces amyloid deposits in brain areas. There is a strong causal association between AD and cardiovascular disease. Several cardiovascular risk factors including hypertension and diabetes are also risk factors for dementia (Stampfer, 2006).

#### Bipolar disorder and schizophrenia

The Wnt pathway and its key enzyme, GSK 3 beta, which antagonizes the canonical Wnt pathway, play an important role in regulating synaptic plasticity, cell survival, and CRs in the mature central nervous system. This pathway is implicated in the pathophysiology and treatment of bipolar disorder (Gould and Manji, 2002; Valvezan and Klein, 2012). GSK3- beta-inhibitor lithium chloride enhances activation of Wnt canonical signaling (Hedgepeth et al., 1997; Sinha et al., 2005; Galli et al., 2013). Lithium activates downstream components of the Wnt signaling pathway *in vivo*, leading to an increase of the beta-catenin protein. GSK3-beta phosphorylates and stabilizes the orphan nuclear receptor Rev-erb alpha, a negative component of the circadian clock. Lithium treatment of cells leads to rapid proteasomal degradation of Rev-erb alpha and activation of clock gene *Bmal1* (Yin et al., 2006). The origin of cyclicity in bipolar disorders has been shown by means of a computational approach, and this disease enters the class of dissipative structures (Goldbeter, 2013). Valproate, an effective medication for the prevention and treatment of mood symptoms in bipolar disorder causes a decrease of PPAR gamma signaling (Lan et al., 2008). Many cardiovascular complications are seen in bipolar disorder (Swartz and Fagiolini, 2012).

An emerging role for Wnt and GSK-3 beta signaling pathways has been found in schizophrenia (Singh, 2013). Sleep and circadian rhythm disruption are seen in schizophrenia (Wulff et al., 2012). Schizophrenia increases risks of cardiovascular disease, particularly coronary heart disease, dyslipidemia, diabetes and hypertension (Hennekens et al., 2005; Andreassen et al., 2013).

### Diseases associated with activation of the Wnt/beta-catenin pathway and decreased expression of PPAR gamma

Numerous diseases present a common denominator: the Wnt/beta-catenin pathway is overexpressed and the PPAR gamma expression is decreased. This explains why type 2 diabetes is commonly associated with hypertension, sympathetic- parasympathetic abnormalities, and cancers and why CR disruptions are often observed among these pathologies. PPAR alpha expression is often increased in these diseases.

#### Impaired sympathetic-parasympathetic system

PPAR gamma and sympathetic nerve activity (SNA) antagonistically regulate energy metabolism and cardiovascular function with the former promoting anabolism and vasorelaxation and the later favoring catabolism and vasoconstriction (Yang et al., 2013). Systemic inactivation of PPAR gamma can be generated constitutively by using Mox2-Cre mice (*MoxCre/flox*) or inducibly by using the tamoxifen system (*EsrCre/flox/TM*). There is an increase in heart rate in both strains of null mice. PPAR gamma deletion causes the activation of SNA. Rosuvastatin increases vascular endothelial PPAR gamma expression and corrects blood pressure variability in obese dyslipemic mice (Desjardins et al., 2008). Sympathetic adrenal function is disrupted in both *Bmal1(-/-)* and *Clock* (mut) mice (Curtis et al., 2007). Although a shorter ultradian rhythm remains, *Bmal1* deletion abolishes the 24-h frequency in cardiovascular rhythms. In humans, heart rate variability has been shown to be driven by an intrinsic mechanism (Hu et al., 2004; Ivanov et al., 2007). CRs and sleep modulate the sympathetic-parasympathetic balance. Sleep deprivation induces a decrease in the global variability, and an imbalance of the autonomous nervous system (ANS) with an increase in sympathetic activity and a loss of parasympathetic predominance. Human individuals homozygous for the longer allele PER3(5/5) compared with PER3(4/4) subjects present an elevated sympathetic predominance and a reduction of parasympathetic activity (Viola et al., 2008). In mice, selective deletion of the *Bmal1* activator PPAR gamma in the vasculature induces a diminution in heart rate circadian variations (Wang et al., 2008). The CCM mouse model exhibits a decrease in heart rate. Conversely, this model does not present differences in systolic, diastolic, and mean blood pressures as compared with controls (Bray et al., 2008).

#### Type 2 diabetes

PPAR alpha activity and its downstream targets are abnormally activated in the diabetic heart, leading to a marked increase in both fatty acid uptake and oxidation (Finck et al., 2005). Chronic activation of the cardiac PPAR alpha pathway which occurs in the diabetic heart, contributes to myocardial lipid accumulation and diabetic cardiomyopathy (Finck et al., 2002). Diabetes alters the circadian clock in the heart. The clock in the heart loses normal synchronization with its environment during diabetes. Diabetes and fasting activate the expression of cardiac FAO. Excessive fatty acid import and oxidation may be a cause of pathological cardiac remodeling in the diabetic heart (Finck and Kelly, 2002). In type 2 diabetes, PPAR alpha is overexpressed and expression of PPAR gamma is deceased. Some TZD PPAR agonists are used to treat type 2 diabetes. The Wnt/beta-catenin signaling pathway is involved in diabetes mellitus (Ip et al., 2012). Expression of PGC-1 alpha is down-regulated in muscles of type 2 diabetic subjects (Liang and Ward, 2006). PGC-1 alpha activates the expression of insulin-sensitive GLUT4 in skeletal muscle and plays a role in preventing insulin resistance and type 2 diabetes mellitus.

The mammalian clock (*Bmal1, Clock, Cry1, Cry2, Per1, Per2, and Per3*) expresses CRs and the phases of these CRs are altered in the hearts from streptozotocin-induced diabetic rats (Young et al., 2002). Two BMAL1 haplotypes are associated with type 2 diabetes and hypertension. This provides evidence of a causative role of *Bmal1* variants in pathological components of the metabolic syndrome (Woon et al., 2007). Rhythmic control of insulin release is deregulated in humans with diabetes. Disruption of the clock components *Clock* and *Bmal1* leads to hypoinsulinemia and type 2 diabetes. Pancreatic islets express self-sustained circadian gene and protein oscillations of the transcription factors CLOCK and BMAL1. The phase of oscillation of the islet genes is delayed in circadian mutant mice, and both *Clock* and *Bmal1* mutants show impaired glucose tolerance, reduced insulin secretion and defects in size and proliferation of pancreatic islets (Marcheva et al., 2010). In rodent models of type II diabetes, mean blood pressure is mildly elevated. The elevation in blood pressure is accompanied by changes in the circadian variation of blood pressure as demonstrated in type 2 diabetes (db/db) mice. The daytime fall in blood pressure in mice is significantly blunted in type 2 diabetes db/db mice (Rudic and Fulton, 2009).

#### Hypertension

PPAR gamma in vascular muscle plays a role in the regulation of vascular tone and blood pressure. Thus, mutations in PPAR gamma induce severe hypertension and type 2 diabetes. Transgenic mice with mutations in PPAR gamma in smooth muscle present vascular dysfunction and severe systolic hypertension (Halabi et al., 2008). PPAR gamma ligands lower blood pressure in both animals and humans. PPAR gamma agonist rosiglitazone improves vascular function and lowers blood pressure in hypertensive transgenic mice (Ryan et al., 2004). In mice, after vascular PPAR gamma deletion, circadian variations of blood pressure and heart rate are dampened through a dysregulation of *Bmal1* (Wang et al., 2008). In a null mouse model with specific disruption of PPAR gamma in endothelial cells, PPAR gamma appears to be an important regulator of blood pressure and heart rate mimicking type 2 diabetes, and mediates the antihypertensive effects of rosiglitazone (Nicol et al., 2005). PPAR gamma regulates the renin-angiotensin system activity in the hypothalamic paraventricular nucleus and ameliorates peripheral manifestations of heart failure (Yu et al., 2012). Activation of PPAR gamma down-regulates the renin-angiotensin system. PPAR gamma is expressed in key brain areas involved in cardiovascular and autonomic regulation. Activation of central PPAR gamma reduces sympathetic excitation and improves peripheral manifestations of heart failure by inhibiting brain renin-angiotensin system activity. PPAR gamma ligands lower blood pressure in both animals and humans, possibly via the PPAR gamma-mediated inhibition of the angiotensin II type 1 receptor expression which results in the suppression of the renin-angiotensin system (Sugawara et al., 2010). Genetic variation in BMAL1 is associated with the development of hypertension in man. BMAL1 dysfunction is associated with susceptibility to hypertension and type 2 diabetes. In conditions of constant darkness, *Cry1/Cry2* deficient mice are hypertensive in the daytime (Rudic and Fulton, 2009). Targeted deletion of *Bmal1* in mice *(Bmal1-KO)* abolishes the CR in blood pressure. Mice with targeted deletion of *PPAR gamma* in the endothelium (*EC-PPAR gamma-KO*) exhibit a striking phenotypic resemblance to endothelial cell (EC)-specific deletion of *Bmal1 (EC-Bmal1-KO)*. The loss of PPAR gamma in the aorta of both *EC-PPAR gamma-KO* mice leads to reduced expression of *Bmal1, Cry1, Cry2*, and *Per2*. The ability of PPAR gamma to modulate blood pressure arises in part from its ability to transactivate *Bmal1*.

#### Atherosclerosis

Wnt/beta-catenin signaling plays a key role in atherosclerosis (Wang et al., 2002). Besides Wnt/beta-catenin, GSK3-beta acts as a beta-catenin independent signal, and plays a crucial role in the regulation of cell proliferation and vascular homeostasis. The progression of atherosclerosis is prevented by PPAR gamma ligands in both animals and humans (Sugawara et al., 2010). Monocyte adhesion to vascular endothelium is one of the early processes in the development of atherosclerosis (Lee et al., 2006). Activation of the canonical Wnt/beta-catenin pathway enhances monocyte adhesion to endothelial cells.

#### Cardiac-restricted overexpression of PPAR alpha (MHC-PPAR)

In mice with cardiac-restricted overexpression of PPAR alpha *(MHC-PPAR*), the expression of PPAR alpha target genes is increased whereas that of genes involved in glucose transport and utilization is repressed (Finck et al., 2002). The metabolic phenotype *of MHC-PPAR* mice mimics that of the diabetic heart. *MHC-PPAR* hearts exhibits profiles of diabetic cardiomyopathy including ventricular hypertrophy, activation of gene markers of pathological hypertrophic growth, and systolic ventricular dysfunction. Transgenic mice overexpressing PPAR alpha in muscle (*MCK-PPAR alpha* mice) developed glucose intolerance. Skeletal muscle of *MCK-PPAR alpha* mice exhibits increased FAO rates and reduced insulin-stimulated glucose uptake. The effects on muscle glucose uptake imply transcriptional repression of the *GLUT4* gene.

#### Aging

Aging is associated with various heart diseases, and this may be attributable, in part, to the prolonged exposure of the heart to cardiovascular risk factors. However, aging is also associated with heart disorders such as diastolic dysfunction that are not necessarily linked to the risk factors for cardiovascular diseases. A mechanistic link between Wnt signaling and premature aging or aging-related phenotypes has been demonstrated (Naito et al., 2010). Tissues and organs from klotho-deficient animals showevidence of increased Wnt signaling. Both *in vitro* and *in vivo*, continuous Wnt exposure triggers accelerated cellular senescence. Thus, klotho appears to be a Wnt antagonist (Brack et al., 2007; Liu et al., 2007b). Specific mutations in the human gene encoding lamin A cause premature aging. In mice and humans, these mutations affect adult stem cells by interfering with the Wnt signaling pathway (Meshorer and Gruenbaum, 2008). Overexpression of *Per* in the fruit fly *Drosophila melanogaster* enhances long-term memory, while in *Per* null flies memory is impaired. This supports a link for circadian genes in the processes of learning and memory (Sakai et al., 2004). In aged animals, the normal photonic stimulation of *Per1* expression is reduced. The free-running period of *Per1*–*luc* rhythmicity is shortened in aged animals and the amplitude of *Clock* and *Bmal1* expression is decreased (Kolker et al., 2003).

#### Neurodegenerative diseases

The common denominator overexpression of the Wnt/beta-catenin pathway and the consequent decrease in PPAR gamma expression play a central role in numerous neurodegenerative diseases (Clevers, 2006a; MacDonald et al., 2009; Yang, 2012). PPAR gamma agonists could potentially inhibit neuro-inflammation and subsequently neurodegeneration. This may partially occur through the ability of PPAR: RXR heterodimers to antagonize *NF_κ_B* mediated gene transcription of several inflammatory mediators such as COX-2, iNOS, and various proinflammatory cytokines. It is not surprising that abnormalities of the cardiovascular system and CRs dysfunction are often associated with neurodegenerative pathologies. Sleep disturbances may predict manifestation of neurodegenerative diseases (Postuma and Montplaisir, 2009).

#### Huntington disease (HD)

HD is a dominantly inherited cytosine-adenine-guanine (CAG) repeat disorder with expanded polyglutamine (polyQ) tracts in huntingtin, causing striatal and cortical degeneration (Walker, 2007). Huntingtin interacts with beta-catenin, beta -TrCP, and axin. Normal huntingtin acts as a scaffold protein, promoting the beta-catenin degradation by facilitating the recognition of beta-catenin by beta -TrCP within the destruction complex (Godin et al., 2010). The binding of beta-catenin to the destruction complex is altered in HD. The presence of an abnormal polyQ expansion in mutant huntingtin leads to a decreased binding to beta-catenin therefore impairing the binding of beta-catenin to the destruction complex and subsequently resulting in beta-catenin accumulation into the cytosol. Thus, beta-catenin levels are up-regulated in HD. Mutant huntingtin alters the stability and levels of beta-catenin. Reducing the canonical Wnt signaling pathway confers protection against mutant huntingtin toxicity in Drosophila (Dupont et al., 2012). Knockdown of Wnt ligands improves the survival of HD flies. Overexpression of armadillo/beta-catenin destruction complex component (AXIN, APC2, or GSK3-beta) increases the lifespan of HD flies.

Early-onset of cardiovascular disease is the second leading cause of death in HD patients. Due to the ubiquitous expression of huntingtin, all cell types with high energetic levels can be impaired. Expression of mutant huntingtin induces cardiac dysfunction in the transgenic model of HD (line R6/2). R6/2 mice develop cardiac dysfunction with cardiac remodeling (e.g. hypertrophy, fibrosis, apoptosis, beta1 adrenergic receptor down-regulation) (Mihm et al., 2007). R6/1 transgenic mice exhibit profound autonomic nervous system-cardiac dysfunction involving both sympathetic and parasympathetic systems, leading to cardiac arrhythmias, and sudden death (Kiriazis et al., 2012). A baroreceptor reflex dysfunction has been described in the BACHD mouse model of HD (Schroeder et al., 2011). Several studies report dysfunction of the autonomic nervous system in HD patients. This may contribute to the increased incidence of cardiovascular events in this patient population that often leads to death. There is a blunted response of the baroreceptor reflex as well as a significantly higher daytime blood pressure in BACHD mice compared to WT controls, which are both indications of autonomic dysfunction. In humans, autonomic dysfunction is present even in the middle stages of HD and affects both the sympathetic and parasympathetic systems (Andrich et al., 2002). Sleep and wake regions of the brain including the brainstem, thalamus, hypothalamus, and cortex are also affected in HD (Kremer et al., 1991). The SCN pacemaker is functional in HD mouse models, so a dysfunction of the circadian circuitry has been proposed to contribute to circadian abnormalities (Pallier and Morton, 2009). Central and peripheral clock gene expression is altered (Maywood et al., 2010). The sleep/wake cycle is disrupted in HD patients characterized by sleep fragmentation at night and delayed sleep phase (Aziz et al., 2010).

#### Amyotrophic lateral sclerosis (ALS)

ALS is a neurodegenerative disease resulting in the progressive loss of upper and lower limb motoneurons and leading to gradual muscle weakening ultimately causing paralysis and death. The Wnt/beta-catenin pathway plays a role in the neurodegeneration of motor neurons in an *in vitro* model of ALS (Pinto et al., 2013). In ALS, a potentially therapeutic pathway may be the activation by PPAR gamma agonists due to their ability to block the neuropathological damage caused by inflammation (Kiaei, 2008). The neuroprotective effect of pioglitazone has been demonstrated in G93A SOD1 transgenic mouse model of ALS and shows a significant increase in their survival. In ALS, PPAR gamma controls natural protective mechanisms against lipid peroxidation (Benedusi et al., 2012).

*Ataxin-2* gene (*ATX2*) is linked to a number of neurodegenerative disorders in humans including ALS and Parkinson disease (PD). ATX2 protein inhibits the production of certain proteins and plays a crucial role in the control of the circadian sleep/wake cycle. *ATX2* regulates the expression of the circadian protein Per in Drosophila. By reducing expression of *ATX2* in Drosophila, the flies are active two and half hours longer. Patients suffering from a form of the neurodegenerative disease spinocerebellar ataxia caused by *ATX2* mutations also experience rapid eye movement sleep disruptions. *ATX2* is necessary for PER accumulation in circadian pacemaker neurons and thus determines period length of circadian behavior. ATX2 is required for the function of TWENTY-FOUR, an activator of PER translation. In humans with ALS, CR of cortisol is impaired (Patacchioli et al., 2003). Both sympathetic and parasympathetic dysfunctions are observed in ALS (Druschky et al., 1999). There are sleep-wake disturbances in patients with ALS (Lo Coco et al., 2011). In human ALS, heart failure is a frequent common cause of death (Gdynia et al., 2006).

#### Parkinson disease (PD)

In a mouse model of PD, a cross talk between inflammatory and Wnt/beta-catenin signaling pathways is involved (L'Episcopo et al., 2012). The Wnt1 regulated Frizzled-1/beta-catenin signaling pathway controls the mesencephalic dopaminergic neuron-astrocyte crosstalk (L'Episcopo et al., 2011). The PPAR gamma agonist pioglitazone modulates inflammation and induces neuroprotection in PD monkeys (Swanson et al., 2011) and mice (Schintu et al., 2009). Expanded glutamine repeats of the ATX2 protein have been identified in fronto-temporal lobar degeneration in PD (Ross et al., 2011). Moreover, a peripheral molecular clock, as reflected in the dampened expression of the clock gene *Bmal1* in leukocytes is altered in PD patients (Cai et al., 2010). There is a disappearance of CRs in a PD dog model (Hineno et al., 1992). Sleep disturbances in PD may be related to CR dysfunction (Hack et al., 2014). Sleep complaints are present in almost half of PD patients. PD patients exhibit increased sleep latency and reduced sleep efficiency. In PD, there is a sustained elevation of serum cortisol levels, reduced circulating melatonin levels, and altered *Bmal1* expression (Breen et al., 2014). PD causes dysfunction of the diurnal autonomic cardiovascular regulation. This dysfunction is profound in patients with severe PD (Haapaniemi et al., 2001).

#### Multiple sclerosis (MS)

Wnt signaling is involved in the MS pathogenesis (Yuan et al., 2012). Mice with experimental autoimmune encephalomyelitis (EAE) have been widely used as a MS model with central nervous system demyelination, neuro-inflammation, and motor impairments. Wnt3a, a Wnt ligand for the canonical pathway, is significantly increased in the spinal cord dorsal horn (SCDH) of the EAE mice. Beta-catenin is also significantly up-regulated. Wnt signaling pathways are up-regulated in the SCDH of the EAE mice and aberrant activation of Wnt signaling contributes to the development of EAE-related chronic pain. PPAR gamma agonists modulate the development of experimental EAE (Drew et al., 2008). Moreover, the risk of myocardial infarction, stroke, heart failure, and atrial fibrillation or flutter is increased in MS patients (Jadidi et al., 2013).

#### Friedreich ataxia (FRDA)

FRDA is a debilitating, life-shortening, degenerative neuromuscular disorder, due to frataxin (FXN) deficiency. FRDA is characterized by neuronal degeneration and heart failure, which are due to loss of transcription of the *FXN* gene caused by a trinucleotide repeat expansion. FXN is a mitochondrial protein involved in iron–sulfur-cluster biogenesis, serving to bind and transfer iron to key electron transport complexes and cytochrome C. Diabetes mellitus and serious heart dysfunction (hypertrophic cardiomyopathy) are associated in most cases. The PPAR gamma agonist Azelaoyl PAF increases FXN protein and mRNA expression in human neuroblastoma cells SKNBE and in primary fibroblasts from skin biopsies from FRDA patients. This offers new implications for the FRDA therapy (Marmolino et al., 2009). It has been shown a coordinate dysregulation of the PPAR gamma co-activator PGC-1 alpha and transcription factor Srebp1 in cellular and animal models of FXN deficiency, and in cells from FRDA patients. A genetic modulation of the PPAR gamma pathway affects FXN levels *in vitro*, supporting PPAR gamma as a new therapeutic target in FRDA (Coppola et al., 2009).

#### Colon cancer

Activation of beta-catenin-Tcf signaling has been observed in colon cancer (Morin et al., 1997). Activation of the Wnt signaling pathway via mutation of the *APC* gene is a critical event in the development of colon cancer (Najdi et al., 2011). Inherited mutations in *APC* lead to the development of non-invasive colonic adenomas (polyps). Wnt pathway activation is a driving force in the development of adenomas. Activation of the Wnt/beta-catenin signaling pathway decreases PPAR gamma activity in colon cancer cells (Jansson et al., 2005) and a loss-of-function mutations in PPAR gamma is associated with human colon cancer (Sarraf et al., 1999).

Colorectal cancer is linked to CR dysregulation (Savvidis and Koutsilieris, 2012). Down-regulation of *Per2* increases beta-catenin protein levels and its target cyclin D, leading to cell proliferation in colon cancer cell lines and colonic polyp formation. *Per2* gene activation suppresses tumorigenesis in colon by down-regulation of beta-catenin. Increased beta-catenin affects the circadian clock and enhances PER2 protein degradation in colon cancer. Suppression of human beta-catenin expression inhibits cellular proliferation in intestinal adenomas. Disruption of the peripheral intestinal CRs may contribute to intestinal epithelial neoplastic transformation of human colorectal cancer. The circadian expression of dihydropyrimidine dehydrogenase, an enzyme that is implicated in the metabolism of the anticancer drug 5-fluorouracil, may be regulated by *Per1* in high-grade colon tumors. The ephrin-Eph cell pathway is linked to the Wnt/beta catenin pathway and is involved in colon cancer (Clevers, 2006a,b).

Functional bowel disorders are associated with autonomic disturbance (Tougas, 2000). People with type 2 diabetes have an increased risk of developing colorectal cancer. Diabetes is associated with a higher risk of colon cancer (Yuhara et al., 2011). Heart disease increases at twice the risk of bowel cancer. Colon cancer and coronary artery disease are known to share similar risk factors (smoking, high-fat diet, obesity, diabetes, high blood pressure, and sedentary lifestyle) which increase the risk of colon cancer. This suggests that the two diseases may be connected (Chan et al., 2007). People with coronary artery disease are more likely to develop colon cancer than those without.

## Synthesis

Circadian rhythms (CRs) are particularly fascinating phenomena. They go very far back in evolution. The existence of a CR is the signature of instability. Beyond a point of bifurcation, an unstable thermodynamic system can evolve spontaneously into a periodic state. These periodic oscillations correspond to a phenomenon of self-organization in time and have been called “dissipative structures” (Prigogine et al., 1974). Dissipative structures are far-from-equilibrium systems, such as cyclones, hurricanes, lasers, Bénard cells, Belousov–Zhabotinsky reactions, Turing structures, circadian rhythms, and more generally most of the living organisms. CRs are based on the existence of negative feedback loops. Oscillatory behavior gradually has been integrated into the living world to become one of its major characteristics. In the cardiovascular system, circadian genes show properties of anticipation and this makes it possible to coordinate lipid and carbohydrate metabolism with the cardiovascular function, especially for blood pressure and heart rate. Dysfunction of CRs can be associated with serious clinical problems and may induce a negative impact on quality of life, sometimes with a poor prognosis. Abnormalities of circadian gene function may result in the occurrence of metabolic syndrome, obesity, and even more seriously, stroke, or myocardial infarction.

Two major systems interfere with circadian genes, namely the canonical Wnt pathway, and the PPAR system. In some cases of diseases presenting profiles 1 or 2, there is not always evidence of the exact influence of CRs on the Wnt-beta catenin-PPAR gamma pathway and cardiac function. PPAR gamma controls the circadian *Clock-Bmal1* genes in the vascular system. Importantly, there is an opposite between activation of the Wnt pathway and PPAR gamma. This is attested by their respective profiles in numerous diseases, either cardiovascular diseases or pathologies with cardiovascular complications. There is a subtle thermodynamic regulation of CRs that run far-from-equilibrium; moreover, there is the need to maintain the balance between the two systems canonical Wnt and PPAR gamma. Indeed, activation of the Wnt system with inactivation of PPAR gamma favors diabetes, hypertension, several cancers, and neurodegenerative diseases. The reverse is observed in ARVD, osteoporosis, Alzheimer disease, bipolar disorder, schizophrenia, and myocardial ischemia. The extreme complexity of the Wnt-PPAR systems and their numerous inter-related pathways partly explain their involvement in numerous diseases. We remain surprised by both the number and the importance of these diseases, causing considerable morbidity, and mortality and heavy social and economic costs.

The discovery and use of new agonist or antagonist pharmacological agents acting on PPARs, and more generally, directly or indirectly implied in the canonical Wnt system, are particularly important. This leads to numerous novel therapeutic approaches. PPAR gamma is a key regulator of lipid metabolism and its activation by some TZD is used for the treatment of type 2 diabetes and protects against atherosclerosis. However, some TZD have been reported to cause a higher rate of fractures in human patients. Pharmacological inhibition of PPAR gamma represents a potential therapeutic approach for age-related bone loss. Induction of the Wnt pathway or inhibition of Wnt antagonists may offer therapeutic opportunities in treating bone disorders, including osteoporosis. Antibodies targeting the Wnt inhibitor sclerostin lead to increased bone mineral density in post-menopausal women. Lithium, often used to treat bipolar disorder, blocks a Wnt antagonist, decreasing the patient's risk of fractures. Lithium exerts effects on components of the Wnt signaling pathway. The Wnt signaling pathway plays an important role in the treatment of bipolar disorder. The future development of selective GSK3- beta inhibitors may have considerable utility not only for the treatment of bipolar disorder but also for a variety of neurodegenerative disorders. Therapies targeting the Wnt pathway are not without risk, and may lead to over-activation of Wnt/catenin and its association with many tumors. However, it is conceivable that targeting Wnt inhibitors may predispose the individuals to tumorigenic phenotypes.

### Conflict of interest statement

The authors declare that the research was conducted in the absence of any commercial or financial relationships that could be construed as a potential conflict of interest.

## Abbreviations

CR: circadian rhythm
PPAR: Peroxisome proliferator-activated receptor
SCN: suprachiasmatic nucleus
APC: adenomatous polyposis coli
Dsh: Disheveled
GSK3-beta: glycogen synthase kinase 3-beta
LRP5/6: low density lipoprotein receptor-related protein 5/6
Fzd: Frizzled
Tcf/Lef: T cell/lymphoid-enhancing binding
Clock: Circadian locomotor output cycles kaput
Ccm: cardiomyocyte-specific clock mutant
FAO: fatty acid oxidation
RXR: retinoid X receptor
PPRE: peroxisome proliferator response element
TZD: thiazolidinedione
Bmal1: Brain and muscle aryl-hydrocarbon receptor nuclear translocator-like
Cry: cryptochrome
Per: Period
Ccg: clock-controlled genes
Ror: related orphan receptor
TZD: thiazolidinediones
ARVC: arrhythmogenic right ventricular cardiomyopathy
DSG: desmoglein
DSC: desmocollin
PKP: plakophilin
PG: plakoglobin
DSP: desmoplakin
PDK: pyruvate deshydrogenase kinase
HIF1 alpha: hypoxia-inducible factor 1 alpha
PGC-1 alpha: PPAR gamma co-activator 1 alpha
M-CPT1: muscle carnitine palmitoyltransferase 1
ANS: autonomous nervous system
AD: Alzheimer Disease
HD: Huntington disease
ALS: Amyotrophic lateral sclerosis
PD: Parkinson disease
MS: Multiple sclerosis
FRDA: Friedreich ataxia
Abeta: amyloid-beta
FXN: Frataxin.
